# Inflammatory cytokines and specific factors influencing lung cancer progression

**DOI:** 10.1016/j.cpt.2025.04.002

**Published:** 2025-04-14

**Authors:** Md. Shalahuddin Millat, Md. Mahmudul Hasan, Mohammad Sarowar Uddin, Md. Abdus Salam, Md. Abdul Aziz, Irin Akhter, Md. Saddam Hussain, Nor Mohammad, Farjana Afrin Tanjum, Md. Saqline Mostaq, Md. Ashiq Mahmud, Mohammad Nurul Amin, Mohammad Safiqul Islam

**Affiliations:** a Department of Pharmacy, Noakhali Science and Technology University, Noakhali, 3814, Bangladesh; b Department of Clinical Pharmacy and Pharmacology, Faculty of Pharmacy, University of Dhaka, Dhaka, 1000, Bangladesh; c Department of Pharmacy, University of Chittagong, Chittagong, 4331, Bangladesh; d College of Pharmacy, University of Manitoba, 750 McDermot Ave, Winnipeg, Canada; e Department of Chemistry, University of Chittagong, Chitagong, 4331, Bangladesh; f Chattogram Maa-o-Shishu Hospital Medical College, Chattogram, 4100, Bangladesh; g School of Basic Pharmaceutical and Toxicological Sciences, University of Louisiana at Monroe, Louisiana, 71201, USA

**Keywords:** Lung cancer, Inflammation, Cytokines, Angiogenesis, Tumorigenesis

## Abstract

Lung cancer (LC) is one of the leading causes of cancer-related morbidity and mortality worldwide. Inflammation is a driver of cancer initiation and progression, affecting processes such as angiogenesis, antiapoptotic pathways, and DNA adduct formation. Cytokines are small proteins that can accelerate or slow tumor growth by controlling associated signaling processes such as cell proliferation, metastasis, and apoptosis. This review reveals the role of tumor necrosis factor-alpha (TNF-α), interferon-gamma (IFN-γ), transforming growth factor-beta (TGF-β), and interleukins in LC. Macrophages play a role in non-small cell lung cancer (NSCLC) pathogenesis and are associated with poor prognosis. A nested case–control study revealed that elevated concentrations of IL-6 and IL-8 were strongly associated with the risk of LC. Specifically, the odds ratio (OR) for IL-6 and IL-8 in former smokers (fourth quartile *vs.* first quartile) was 2.70 (95% confidence interval [CI], 1.55–4.70) and 2.83 (95% CI, 1.18–6.75), respectively. Because C-reactive protein levels are elevated in patients with NSCLC with larger and higher-grade tumors, CRP has been identified as a systemic indicator of chronic inflammation. Insulin-like growth factors influence cellular signal transduction pathways and contribute to tumorigenesis. Soluble tumor necrosis factor receptors have been explored for their role in NSCLC prognosis, highlighting their association with chromogranin. Transient receptor potential cation channel, subfamily M, member 7 (TRPM7), urokinase plasminogen activator, matrix metalloproteinases, and monocyte chemoattractant protein-1 have been identified with a focus on their expression patterns and prognostic significance in LC tissues. Moreover, lung angiogenesis induces vascular endothelial growth factor, soluble intercellular adhesion molecule-1, myeloperoxidase, and tissue inhibitors of metalloproteinase expressions. In conclusion, this review thoroughly summarized the inflammatory cytokines and specific factors influencing LC, providing the basis for further research on potential treatment approaches.

## Highlights

• Inflammation is the body's normal response to injury or infection.
• Cytokines like tumor necrosis factor-alpha (TNF-α), interferon-gamma (IFN-γ), and interleukin (IL) regulate inflammation and immune function.
• Chronic inflammation increases the risk of lung cancer (LC) and promotes tumor growth.
• Targeting cytokines such as IL-6 and TNF-α may offer better therapies for LC.
• Immune modulation through cytokine inhibition could reduce tumor-associated inflammation and enhance treatment efficacy.

## Graphical abstract

**Figure:**
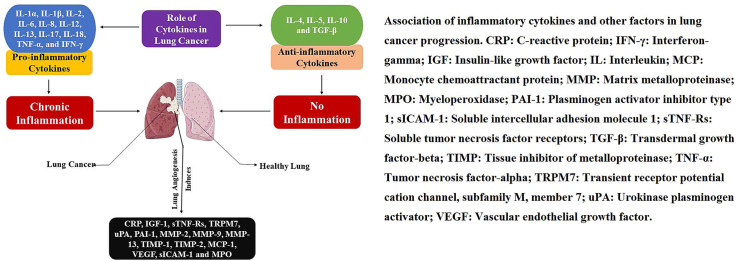

## Introduction

Lung cancer (LC) is a leading cause of cancer-related morbidity and mortality worldwide.^1^ Psychological, environmental, and hereditary risk factors contribute to tumor growth and influence patients’ ability to respond to treatment. These factors increase the risk of developing LC.^2^ Approximately 85–90% of LC cases are non-small cell lung cancers (NSCLCs), which comprise three distinct subgroups: carcinoma of the squamous cell, adenocarcinoma (AC), and large cell carcinoma, accounting for 25–30%, 40%, and 10–15% of LC cases, respectively. Small cell lung cancer (SCLC), the second most common type of LC, accounts for 10–15% of all cases. Apart from the two major types of LC, other tumors can grow in the lungs, such as carcinoid tumors, which account for 1–2% of all cancer cases. NSCLCs are treated with a combination of surgery and adjuvant therapy, whereas SCLCs exhibit aggressive behavior and are typically treated non-surgically.^3^ The most extensive international statistical research indicates that 1.6 million people worldwide died from NSCLC in 2012, while approximately 1.8 million new cases were diagnosed.^4^ Another study revealed millions of new cases and deaths due to LC in 2008.^5^

Cancer is a complex and multifaceted condition involving numerous contributing variables.^6^ Proliferative signaling persistence, growth repressor evasion, immortality via replication, invasion and metastasis activation, angiogenesis induction, and resistance to cell death are the key features of cancer.^7^ According to epidemiological studies, chronic inflammation contributes to >25% of cancer-related deaths.^8^ Beyond the six hallmarks of cancer proposed by Allin et al.,^9^ inflammation has been recognized as a seventh hallmark or an “associated trait” of cancer.^10^^,^^11^ Chronic inflammation and cancer have a reciprocal relationship; tumor cells can initiate a provocative environment and broader host immune response, while persistent inflammation can also facilitate carcinogenesis. Determining the biomarkers involved in cancer progression is crucial because it may aid the identification of high-risk patients via screening programs, which may help prevent cancer or detect it sufficiently early for successful treatment. Serum levels of leukocytes, fibrinogen, and C-reactive proteins (CRPs) are frequently identified as biomarkers of systemic inflammation.^9^ Numerous studies have evaluated each of these markers separately; however, only a few have examined their overall association with LC.

Cytokines are small protein molecules that participate in inflammatory processes, some of which may be crucial for tumor development, invasion, and metastasis. TNFs, ILs, chemokines, IFNs, and lymphokines are examples of such molecules. Cytokines influence the immune system function, inflammatory reactions, maturation, proliferation, and reactivity of specific cell types. Cytokines can be classified as pro and anti-inflammatory. Cytokines exert functional effects on LC progression. Furthermore, their crucial functions in LC-associated signaling systems suggest that some cytokines may play dual roles as prognostic and diagnostic biomarkers.^12^ Many immune and physiological responses are mediated by cytokines, which can induce a wide range of biological effects, including tumor killing. Activated macrophages produce a variety of mediators to control host defenses by promoting immune functions within the cells. In reaction to stimulation, macrophages release oxidants, such as nitric oxide, and pro-inflammatory cytokines, such as TNF-α, IFN-α, IL-1β, IL-6, and IL-12. In contrast, anti-inflammatory cytokines, including IL-10, TGF-β, and IL-11, are generated to control the effects of the macrophages. T-cells are also involved in tumor immune surveillance. Macrophages are triggered by the secretions of T-helper 1 (Th1) cells, which include IL-2, IFN-γ, and TNF-α. Moreover, IL-4, IL-5, IL-10, and IL-13 are secreted by Th2 cells and can stimulate and inhibit certain macrophage functions.^13^

The urokinase plasminogen activator (uPA) system comprises uPA, urokinase plasminogen activator receptor (uPAR), and plasminogen activator (PA) inhibitor type 1 and 2 (PAI-1 and PAI-2). Certain entities capable of producing uPA include tumor cells, fibroblasts, monocytes, and epithelial cells. One particular receptor, uPAR, is activated by uPA and produces a variety of effects, such as metastasis, cell adhesion, activation of signaling networks, and activation of PA. Although PAI-1 and PAI-2 are both specific uPA inhibitors, PAI-1 functions more effectively than PAI-2 and is the primary uPA inhibitor. Poor prognosis is strongly linked to the manifestation of uPA, uPAR, PAI-1, and PAI-2.^14^ The key tissue-specific mediators of matrix disintegration are matrix metalloproteinases (MMPs). The most prevalent, MMP-2 (gelatinase A), is constitutively released by most cells, particularly endothelial and epithelial. Activated connective tissue and inflammatory cells produce MMP-9 (gelatinase B). In numerous human malignancies, both enzymes are found in surrounding neoplastic, stromal, and inflammatory cells. Tissue inhibitors of metalloproteinases (TIMP-1 and TIMP-2), which are naturally occurring tissue inhibitors of gelatinases, have been studied in various histological forms of LC cell lines, and their gelatinolytic actions are closely correlated with the metastatic progression of SCLC.^14^ Furthermore, a recent study has indicated that MMP-9 and TIMP-1 do not circulate normally in advanced LC.^15^ Transient receptor potential (TRP) proteins that are frequently altered during cancer progression include transient receptor potential vanilloid (TRPV) 2 and 6, TRP melastatin (TRPM) 7, and the canonical TRP proteins (TRPC1 and TRPC6). One of these, TRPM7, has been linked to the malignant predisposition of cancer stem cell subgroups and is abnormally expressed in lung AC and squamous cell carcinoma (SCC).^16^

The superfamily of physiologically active peptides known as chemokines comprises approximately 50 members. Among the chemokines, monocyte chemoattractant protein-1 (MCP-1) and chemokine (C-C motif) ligand 2 (CCL2) are particularly relevant for cancer growth and progression. Several malignancies, such as brain tumors and ovarian, lung, breast, and prostate cancers, have elevated MCP-1 levels. MCP-1 is released by cancerous cells of the breast, which attract monocytes associated with inflammation that generate vascular endothelial growth factor (VEGF) to encourage cancer cell spread and pulmonary metastasis. Several cytokines and growth factors, including TNF-α, IL-1β, IFN-γ, and platelet-derived growth factor (PDGF), can produce MCP-1.^17^

Human malignancies and inflammatory disorders (including coronary artery disease and airway inflammation) have higher levels of soluble intercellular adhesion molecule 1 (sICAM-1), which is generated when the ICAM-1 cell surface is broken by proteases. Several variables, including smoking and the expression of certain cytokines, particularly TNF-ɑ, can affect serum concentrations of sICAM-1. sICAM-1 may facilitate tumor growth by enabling malignant cells to evade immune responses.^18^ A recent study has revealed the relationship between sICAM-1 and LC onset and progression. The correlation between increased serum sICAM-1 levels, poor prognosis, and LC development has been evident in different studies.^19^^,^^20^ However, other studies have failed to identify a statistically significant relationship between LC stage, prognosis, and serum sICAM-1 levels.^21^^,^^22^ Myeloperoxidase (MPO) is also present in the polymorphonuclear leukocytes and neutrophils. Hypochlorous acid and other reactive oxygen species (ROS) are strong antimicrobial chemicals generated by MPO that have potential microbicidal action. Additionally, MPO activates certain procarcinogens such as arylamines, 4-aminobiphenyl, and benzo[a]pyrene precursors. MPO is taken up and released as a component of the inflammatory response following exposure to lung irritants, such as cigarette smoke. There is a link between a single nucleotide change in MPO and a greater likelihood of lung carcinoma.^23^

This review provides a comprehensive comparison of the role of pro-inflammatory and anti-inflammatory cytokines in LC, focusing on their dual roles in tumor progression and immune evasion. Unlike previous studies, this review emphasizes the specific mechanisms by which cytokines influence cancer development and immune responses across various stages of LC. Reviewing the potential involvement of inflammatory cytokines and particular factors in LC is the ultimate objective.

## Inflammatory cytokines and certain factors influencing lung cancer development

Various inflammatory signaling molecules, such as chemokines and cytokines, can promote LC onset. Cytokines can either promote (including IL-1α, IL-1β, IL-2, IL-6, IL-8, IL-12, IL-13, IL-17, IL-18, TNF-α, and IFN-γ) or repress (including IL-4, IL-5, IL-10, and TGF-β) inflammation. Patients with LC also have higher serum levels of various other variables, including CRP, insulin-like growth factor (IGF)-1, soluble tumor necrosis factor receptors (sTNF-Rs), TRPM7, uPA, PAI-1, MMP-2, MMP-9, MMP-13, TIMP-1, TIMP-2, MCP-1, VEGF, sICAM-1, and MPO.^24^ Table 1 shows the correlation between inflammatory cytokines, certain factors, and LC, and Table 2 compares the roles of pro-inflammatory and anti-inflammatory cytokines in LC.

**Table 1:** Association of inflammatory cytokines and specific factors with different histological types of lung cancer.

Inflammatory Cytokines	SCLC	NSCLC	Squamous cell carcinoma	Lung adenocarcinoma	Neuroendocrine lung carcinoma	References
IL-1α	–	+	–	–		22,23
IL-1β	–	**+**	–	–	–	36,37
IL-2	–	**+**	–	–	–	39,40
IL-6	–	+	–	–	–	50
IL-8	**+**	**+**	–	–	–	53,56
IL-12	–	–	–	–	+	58,156
IL-13	–	**+**	–	**+**	–	61,157
IL-17	–	+	–		–	71,157,158
IL-18	+	+	–	–	–	76,159
TNF-α	–	+	–	–	–	78
IFN-γ	–	+	–	–	–	84
IL-4	–	+	+	–	–	88,92,160
IL-5	–	+	–	–	–	93
IL-10	–	+	–	–	–	99,161
TGF-β	–	+	–	–	–	161
CRP	+	+	+	–	–	108,109
IGF-1	–	+	–	–		117
sTNF-Rs	–	+	–	–	–	118
TRPM7	–	–	+	+		16
uPA/PAI-1	–	+	+	+	–	122,123,162
MMP-2	–	+	–		–	163
MMP-9	–	+	–	+	–	14,164
MMP-13	–	+	–	–	–	128
TIMP-1 and TIMP-2	+	+	–	–	–	129
MCP–1	–	+	–	–	–	128
VEGF	+	+	–	–	–	130,132
sICAM-1	+	+	–	–	–	20,22
MPO	–	–	+	+	–	25

(–) means no correlation, (+) means positive correlation. CRP: C-reactive protein; IFN-γ: Interferon-gamma; IGF: Insulin-like growth factor; IL: Interleukin; MCP: Monocyte chemoattractant protein; MMP: Matrix metalloproteinase; MPO: Myeloperoxidase; NSCLC: Non-small cell lung cancer; PAI-1: Plasminogen activator inhibitor type 1; SCLC: Small cell lung cancer; sICAM-1: Soluble intercellular adhesion molecule 1; sTNF-Rs: Soluble tumor necrosis factor receptors; TGF-β: Transdermal growth factor-beta; TIMP: Tissue inhibitor of metalloproteinase; TRPM7: Transient receptor potential cation channel, subfamily M, member 7; TNF-α: Tumor necrosis factor-alpha; uPA: Urokinase plasminogen activator; VEGF: Vascular endothelial growth factor.

**Table 2:** Dual roles of pro-inflammatory and anti-inflammatory cytokines in lung cancer progression.

Cytokine type	Cytokine	Role in lung cancer
Proinflammatory	IL-1α, IL-1β	Promotes angiogenesis, immune evasion, and tumor growth; induces inflammation.^165^
	IL-2	Involved in tumor immunity, it stimulates immunological responses by encouraging T-cell activation and cytotoxic activity.^38^
	IL-6	Enhances angiogenesis, tumor invasion, growth, and metastasis; it additionally fosters chronic inflammation and immune evasion.^166^
	IL-8	Induces immune cells to the tumor microenvironment, which causes angiogenesis, tumor development, and metastasis.^167^
	IL-12	Promotes antitumor immunity by stimulating T-cell and natural killer–cell activity; however, in certain situations, it can also promote tumor growth.^168^
	IL-13	Enhances the tumor microenvironment, which encourages fibrosis and tumor growth, particularly in lung cancer.^169^
	IL-17	Causes inflammation, promotes tumor growth, and prevents apoptosis, all of which are involved in the advancement of lung cancer.^170^
	IL-18	Induces IFN-γ production and enhances immune responses, promoting antitumor immunity; however, it may also foster inflammation.^171^
	TNF-α	Drives chronic inflammation and helps tumor cell survival, proliferation, and metastasis.^172^
	IFN-γ	Enhances immune surveillance but prolonged exposure can foster inflammation, contributing to tumor progression.^173^
Anti-inflammatory	IL-4	Inhibits proinflammatory cytokine production; promotes tumor immune evasion and suppresses antitumor immunity.^174^
	IL-5	Involved in the recruitment of eosinophils, which may promote tumor progression in certain cancer types, though less studied in lung cancer.^175^
	IL-10	Suppresses inflammation and immunological activation, which may make it possible for malignancies to evade infection.^175^
	TGF-β	In advanced stages of cancer, it promotes tumor growth, metastasis, and immune evasion by suppressing inflammation and immunological responses.^176^

IFN-γ: Interferon-gamma; IL: Interleukin; LC: Lung cancer; TGF-β: Transdermal growth factor; TNF-α: Tumor necrosis factor-alpha.

### Interleukin-1α

**Figure 1:**
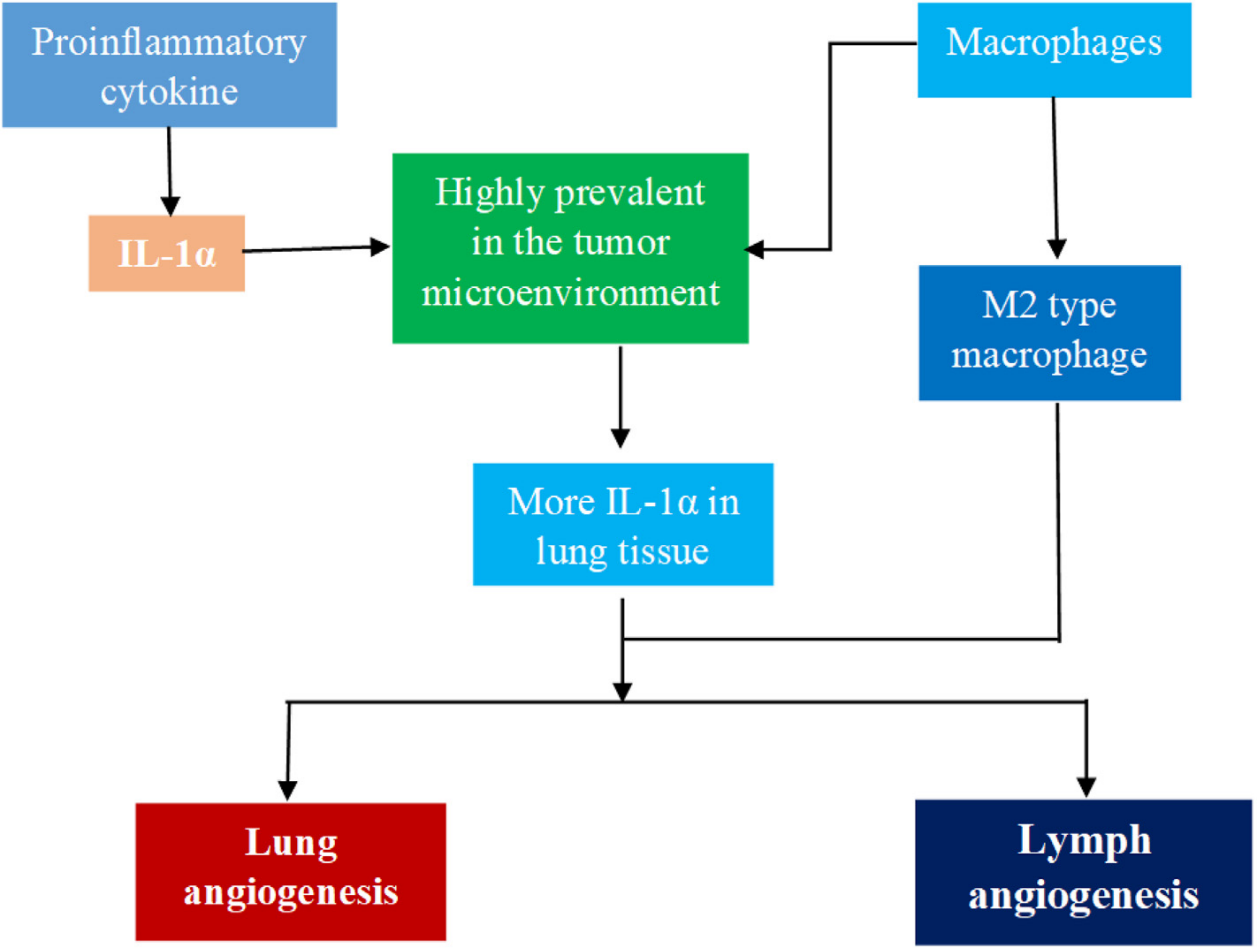
Correlation between IL-1 expression and clinical outcomes in LC patients. High IL-1 levels are associated with increased angiogenesis and lymph angiogenesis in lung tumor tissues. IL-1α: Interleukin-1 alpha; LC: Lung cancer; M2: Alternatively Activated Macrophage Type 2.

Carcinogenesis is strongly associated with inflammation.^11^ IL-1α is a prominent pro-inflammatory cytokine that induces the progression of different types of cancer. IL-1α is a cytokine with dual roles: pro and antitumor effects in certain types of malignancies.^25^ Macrophages are highly prevalent in the tumor microenvironment. In patients with NSCLC, a high correlation between macrophage invasion and poor prognosis has been found, suggesting their involvement in the progression of lung malignancy. When tumor-bearing mice develop LC, the lesions themselves become a source of IL-1α and IL-1β. Cytokines, such as IL-1α, are widely distributed within the lung tumor environment. Epithelial and tumor cells appear to be the principal sources of IL-1α.^26^ Compared with their less cancerous counterparts, highly cancerous human lung carcinoma cell lines secrete more IL-1α and form tumors with better angiogenesis and lymphogenesis. Additionally, M2-type macrophages heavily invade tumors. Angiogenesis and lymph angiogenesis are activated by IL-1α because these effects were reduced by an IL-1R antagonist.^27^ Figure 1 illustrates the potential correlation between IL-1α and LC prognosis.

### Interleukin-1β

**Figure 2:**
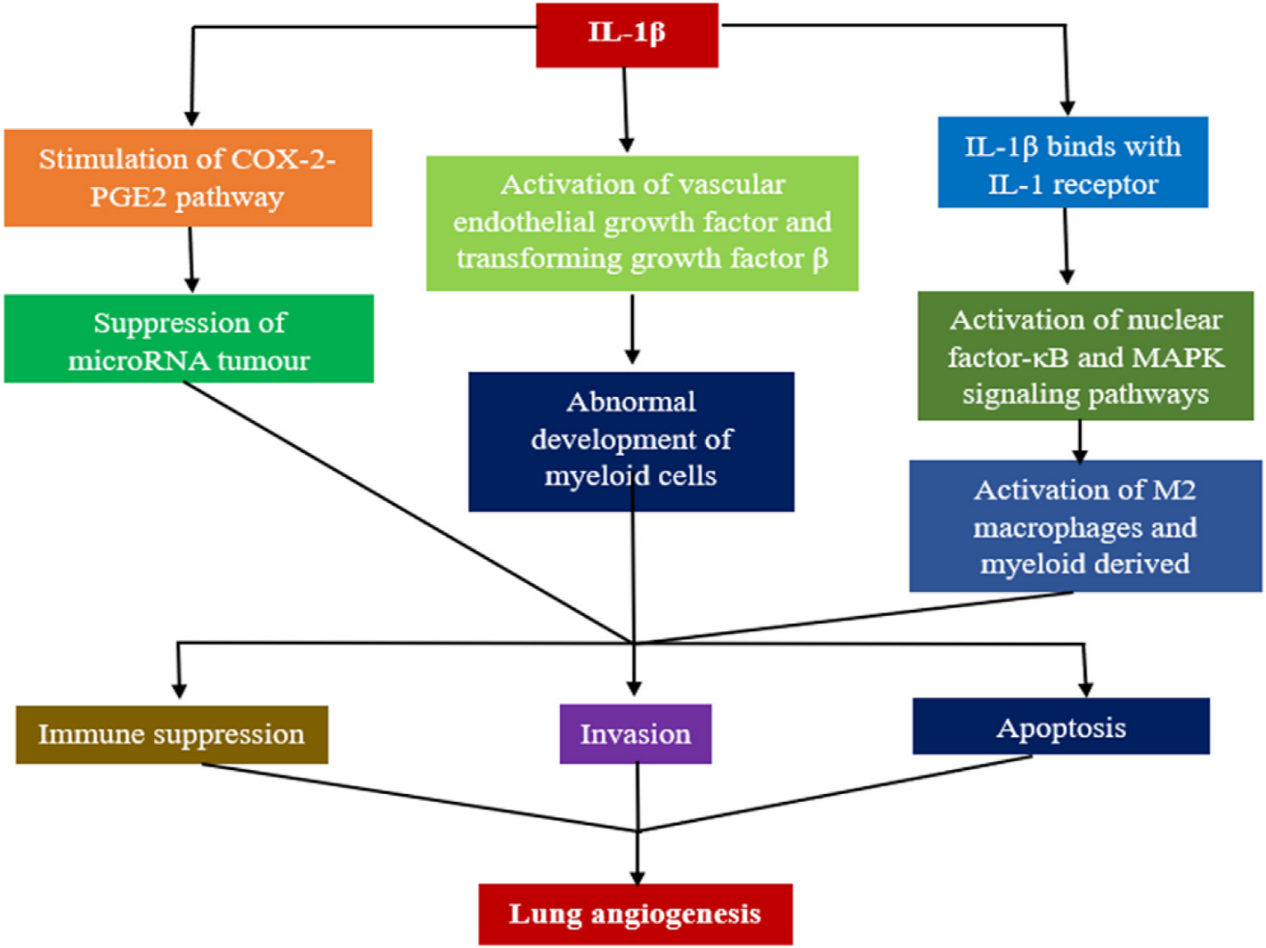
Role of IL-1β in LC progression. IL-1β causes immune suppression of patients, invasion of lung tissue, and resistance to tumor cell apoptosis through several mechanisms and helps lung angiogenesis. IL: Interleukin-1; COX-2: Cyclooxygenase-2; M2: Alternatively Activated Macrophage Type 2; MAPK: Mitogen-activated protein kinase; microRNA: MicroRNA; miR-101: MicroRNA-101; PGE2: Prostaglandin E2.

IL-1β stimulates tumor growth and metastasis by activating pathways that upregulate growth factors, such as TGF-β, prostaglandin (PGE)-2, and VEGF.^27^ *IL-1β* plays a critical role as a mediator at the onset of the inflammatory response in lung illnesses, including lung carcinoma and chronic obstructive pulmonary disease.^28^ Bronchial fibrosis, increased thickness, and cytokine production are signs of lung inflammation that block airflow.^29^^,^^30^ Nod-like receptor protein-3 (NLRP3) found in lung tumors controls pro-tumor activity in macrophages and is associated with lung metastasis.^26^ The nuclear factor-κB (NF-κB) and mitogen-activated protein kinase (MAPK) signaling pathways are activated while IL-1β binds to the IL-1 receptor.^31^ Myeloid-derived suppressor cells (MDSCs) and M2 macrophages are activated in response to granulocyte-macrophage colony-stimulating factors. This causes their recruitment into the intratumoral space, which promotes immune evasion, tumor invasiveness, and malignant growth.^26^^,^^30^, ^31^, ^32^ *IL-1β* promotes abnormal development of myeloid cells, known as leukocytosis, in the spleen.^33^ This inflammatory cytokine potentiates chemokine release and the expression of leukocyte adhesion molecules on the vascular endothelium, resulting in increased angiogenesis, chemotaxis, and cell adhesion.^34^ IL-1β controls the suppression of the immune system, invasion, the epithelial–mesenchymal transition, apoptosis resistance, as well as angiogenesis, which are all triggered by the cyclooxygenase-2-PGE-2 pathway.^35^ After IL-1β-mediated activation of the cyclooxygenase 2 pathway, the microRNA (miRNA) tumor suppressor miR-101 was dramatically reduced in NSCLC cells, suggesting a potential role for IL-1β in LC progression.^36^ A considerably greater risk of NSCLC was linked to the T/T genotype of the *IL-1β-31* gene, as reported by Bhat et al. (*P* = 0.001; odds ratio [OR], 2.8; 95% confidence interval [CI], 1.52–5.26). Patients with LC who have the *IL 1β-*31 TT genotype express more *IL-1β* messenger RNA (mRNA). An environment with more inflammatory triggers is created by the above genotype (*IL-1β*-31 T/T) in the *IL-1β* regulatory area, thereby increasing the probability of lung tumors.^37^ The pathways through which IL-1β is linked to LC progression are shown in Figure 2.

### Interleukin-2

**Figure 3:**
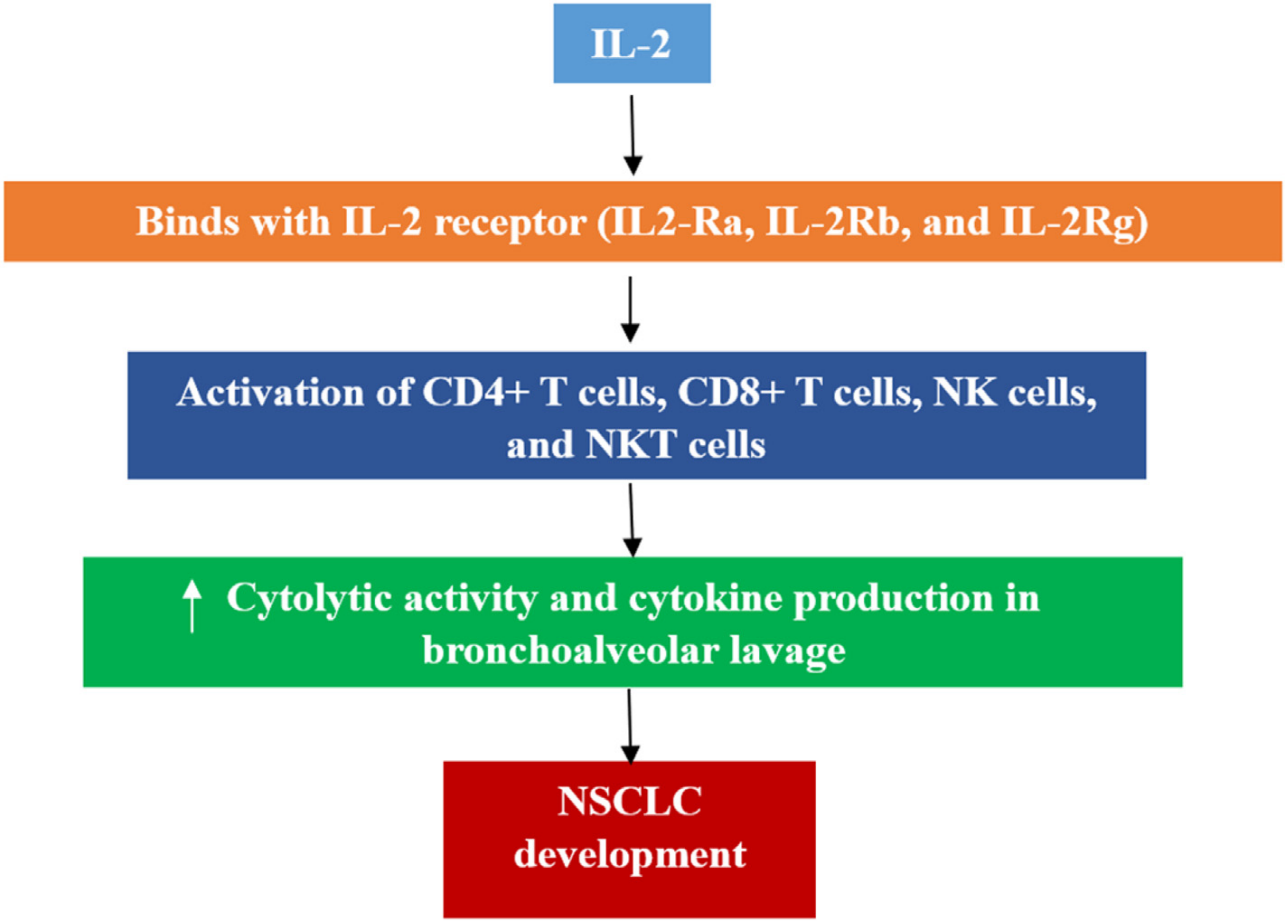
Mechanism of IL-2 in the development of NSCLC. IL-2 over-activates cytolytic activity and upregulates cytokines via the modification of immune cells' response, hence the development of NSCLC. CD: Cluster of differentiation; IL: Interleukin; NK: Natural killer; NKT: Natural killer T cell; NSCLC: Non-small cell lung cancer.

The cytokine IL-2 was first identified around 38 years ago under the name “T cell growth factor.” However, it was later discovered that it stimulated B, natural killer (NK), and natural killer T (NKT) cells in different ways. After stimulation with an antigen, CD4+ T cells, CD8+ T cells, NK cells, and NKT cells release IL-2. The stimulation, proliferation, and regulation of T and NK cell effector activities, including cytolytic function and release of cytokines, along with their capacity to destroy other cells, are all greatly impacted by IL-2. The IL-2 receptor that binds to IL-2 is made up of three subunits: IL-2Ra (CD25), IL-2Rb (CD122), and IL-2Rg (CD132).^38^ Patients with NSCLC have considerably higher serum IL-2 levels than healthy individuals.^39^ Tumor development and IL-2 levels are correlated. IL-2 levels in the urine, blood, induced saliva, and bronchoalveolar lavage (BAL) of patients with NSCLC were greater. IL-2 concentrations in breath condensate, induced sputum, and BAL of patients with NSCLC showed a significant positive correlation (*r* = 0.6, *P* < 0.01). ACs and SCCs did not differ significantly from each other.^40^ Hamid et al. employed IL-2 and anti-programmed death-ligand 1 (PD-L1) inhibitors to improve LC detection and treatment in patients with compromised immune systems.^41^ Based on the hypothesis that antibody cells strengthen the immune response, a mathematical model was developed. The fractal-fractional operator (FFO) was used to convert the model into a fractional-order system. This method also assesses how well IL-2 and anti-PD-L1 produce anticancer cells and slow the spread of LC.^42^ A possible mechanism by which IL-2 induces NSCLC is shown in Figure 3.

### Interleukin-6

Several organs, including activated leukocytes, adipose tissue cells, and endothelial cells, release IL-6, an essential pro-inflammatory cytokine. Poor prognosis in patients with LC is associated with IL-6 expression in premalignant epithelial cells, suggesting a potential link between circulating IL-6 and LC.^43^ Therefore, IL-6 may be important for the growth of malignant tumors. Elevated circulating IL-6 levels are associated with survival in LC^44^ through tumor progression.^45^ Studies have demonstrated that IL-6 and signal transducer and activator of transcription (STAT) are linked to the growth and progression of various cancers, particularly those of the lungs, prostate, kidneys, and breasts.^46^ Recently, we elucidated how IL-6/IL-6 receptor signaling promotes LC cell proliferation using mouse models.^46^ The enhanced effect of cancer-associated fibroblasts (CAFs) on the capacity of lung tumor cells to metastasize is explained by the IL-6/STAT3 signaling system.^47^

**Figure 4:**
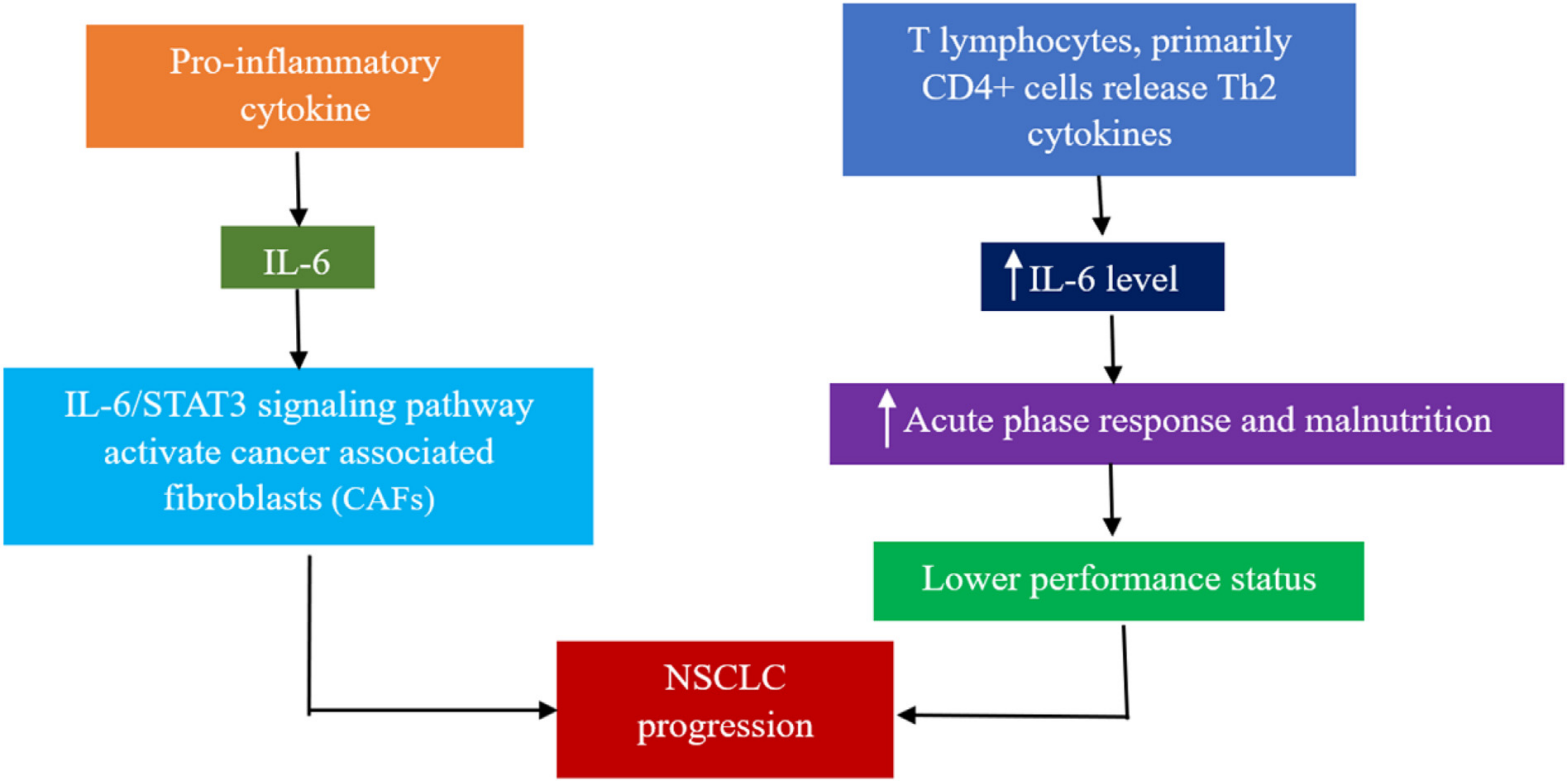
Mechanism of IL-6 in the development of LC. IL-6 activates cancer-associated fibroblast and promotes acute phase response and malnutrition to help the progression of NSCLC. CD: Cluster of differentiation; IL: Interleukin; LC: Lung cancer; NSCLC: Non-small cell lung cancer; Th: T-helper; STAT: Signal transducer and activator of transcription.

The production of cytokines, including IL-6 or IL-1β, by the alveolar macrophages of patients with LC is considerably higher than that of patients with nonmalignant lung illnesses. IL-6 is a protein that tumor cells always produce; other proteins are only produced in response to certain stimuli. High levels of IL-6 in individuals with tumors are caused by an increase in T lymphocyte production, especially CD4+ cells, which release Th2 cytokines. IL-6 further controls the action of lymphokine-activated killer (LAK) cells by enhancing TNF-α release as well as TNF receptor expression.^13^ Malnutrition is prevalent in patients with LC linked with poor survival rates.^48^ Regarding the severity of the acute-phase response, Martin et al. reported increased serum levels of IL-6 in individuals with LC,116 which are associated with acute-phase response, malnutrition, and reduced survival time.^49^ Neuron-specific enolase (NSE), a prominent NE marker, is suppressed by IL-6 in NSCLC-NE cells, and cell proliferation is accelerated.^50^ Circulating IL-6 levels have been proposed as a predictor of survival in patients with NSCLC who receive chemotherapy.^51^ Figure 4 illustrates the mechanism by which IL-6 significantly contributes to LC development.

### Interleukin-8

**Figure 5:**
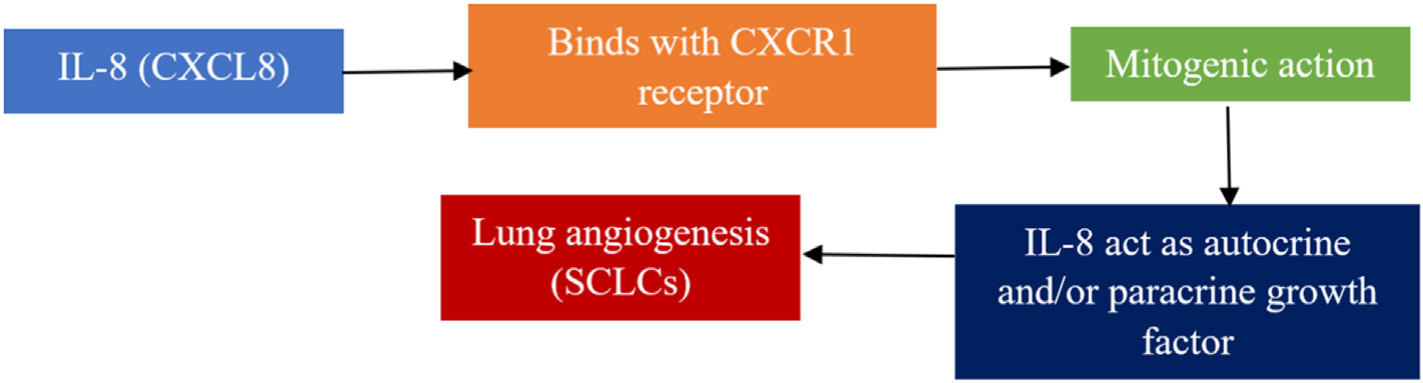
Mechanistic view of IL-8 in LC. By acting on autocrine and paracrine glands, IL-8 stimulates lung angiogenesis and SCLC development. CXCL: C-X-C motif chemokine ligand; CXCR: C-X-C motif chemokine receptor; IL: Interleukin; LC: Lung cancer; SCLC: Small cell lung cancer.

IL-8 belongs to the CXC family. Initially, it was classified as a neutrophil chemoattractant with anti-inflammatory properties.^52^ The molecular mechanism underlying the role of IL-8 in tumor development remains uncertain. IL-8, a potent angiogenic factor, has been linked to metastasis in several malignancies, including NSCLC.^53^^,^^54^ In NSCLC, increased IL-8 levels have been linked to angiogenesis and tumor growth.^52^ Acting as an autocrine or paracrine growth factor for LC cells (SCLCs), the mitogenic action of this chemokine is mostly mediated through CXCR1 receptor lines, which act as autocrine or paracrine growth factors in SCLCs.^55^ Compared with controls, the whole blood of patients with NSCLC had noticeably increased levels of IL-8. In contrast with the controls, LTB-4 and IL-8 were found at higher levels in the whole blood and exhaled breath condensate (EBC)of patients with NSCLC, particularly in smokers and ex-smokers.^56^ Figure 5 shows a possible mechanism by which IL-8 expression is correlated with LC.

Using reverse transcription-polymerase chain reaction (RT-PCR), Zhu et al. demonstrated that IL-8 mRNA was present in all NSCLC cell lines, particularly A549, H460, and MOR/P.^55^ However, Lu165, CORL24, and GLC-19 cell lines did not express IL-8 mRNA, whereas SCLC cell lines such as H69, H345, and H711 showed modest expression of the mRNA. According to Arenberg et al., the growth rates of two distinct human NSCLC cell lines, A549 and Calu-1, in severe combined immunodeficiency (SCID) mice were directly associated with tumor-derived IL-8.^56^ The progression of lung tumors in humans is accelerated by angiogenic IL-8, which does not function as an autocrine growth factor to encourage the growth of NSCLC cells.^56^

### Interleukin-12

The immunocytochemical examination of the tissue distribution of cytokines (IL-2 and IL-12) in neuroendocrine LC was described by Kasprzak et al. in 2003.^57^ They suggested that IL-2 may be involved in the development of malignant cells in lung carcinoids.^57^ Chromogranin A (CgA) and NSE were found in all lung carcinoids, confirming their use as adjunct diagnostic tools for lung neuroendocrine malignancies.^58^ Both markers showed strong responses (scores of 6–12). In every instance, co-expression of IL-2 and IL-12 was also observed. Two patients had low, two had moderate, and six had high IL-2 reaction intensities. In half of the cases, IL-12 expression was moderate, whereas it was high in the other half.^57^

### Interleukin-13

**Figure 6:**
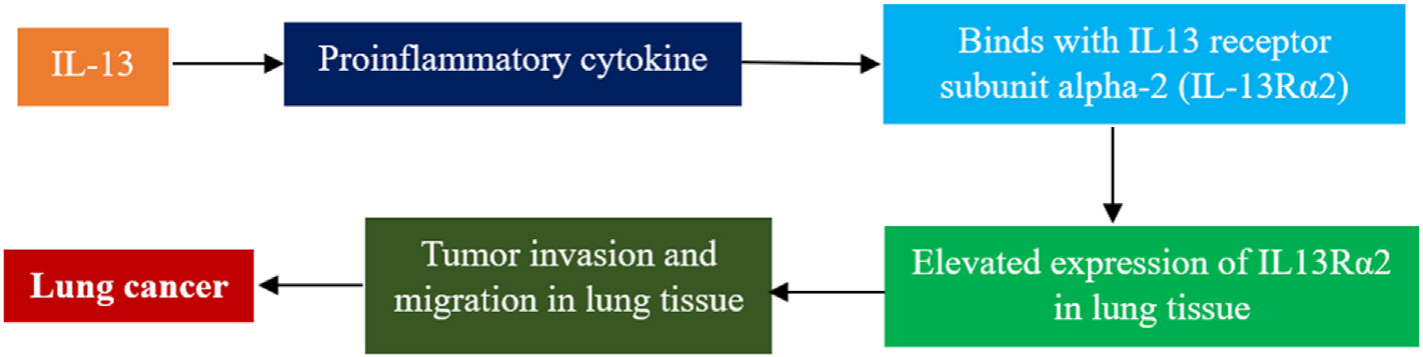
Correlation between IL-13 and LC development. IL-13 promotes tumor invasion and migration in the lung tissue, aiding LC development. IL: Interleukin; IL-13Rα2: IL-13 receptor subunit alpha-2; LC: Lung cancer.

Tumor development, invasion, and metastasis are facilitated by chemokine-mediated inflammation.^57^ ILs, which are pro-inflammatory chemicals, are frequently found in inflammation associated with cancer.^59^ Asthma, autoimmune disorders, and ulcerative colitis are only a few clinical ailments that have been linked to IL-13, a cytokine that induces inflammation and is generated by Th2 cells.^60^ The receptor subunits where IL-13 binds are IL-13 receptor subunit alpha-1 (IL-13Rα1) and IL-13 receptor subunit alpha-2 (IL-13Rα2). Additionally, IL-13Rα2 is a target for cancer treatment and is linked to prognosis in human tumors.^60^ As IL-13 and IL-13Rα2 bind, tumor invasion and migration are accelerated. While nearby normal lung tissues showed little or no IL-13Rα2 expression, IL-13Rα2 expression was primarily associated with LC in humans, and compared with the other histological categories, lung AC and NSCLC had poorer outcomes when IL-13Rα2 was overexpressed. LC, particularly lung AC, may have a biomarker in IL-13Rα2 since it boosts LC cell proliferation and resistance to chemotherapy. SCC, AC, and lung cell carcinoma (LCC) are the three histological subtypes of NSCLC. All histological NSCLC subtypes showed elevated IL-13 mRNA levels, whereas SCC had much higher levels than LCC.^61^ A greater amount of IL-13 mRNA and protein in NSCLC cell lines was confirmed by Huang et al.*^62^* Figure 6 shows a possible link between IL-13 and LC development.

### Interleukin-17

**Figure 7:**
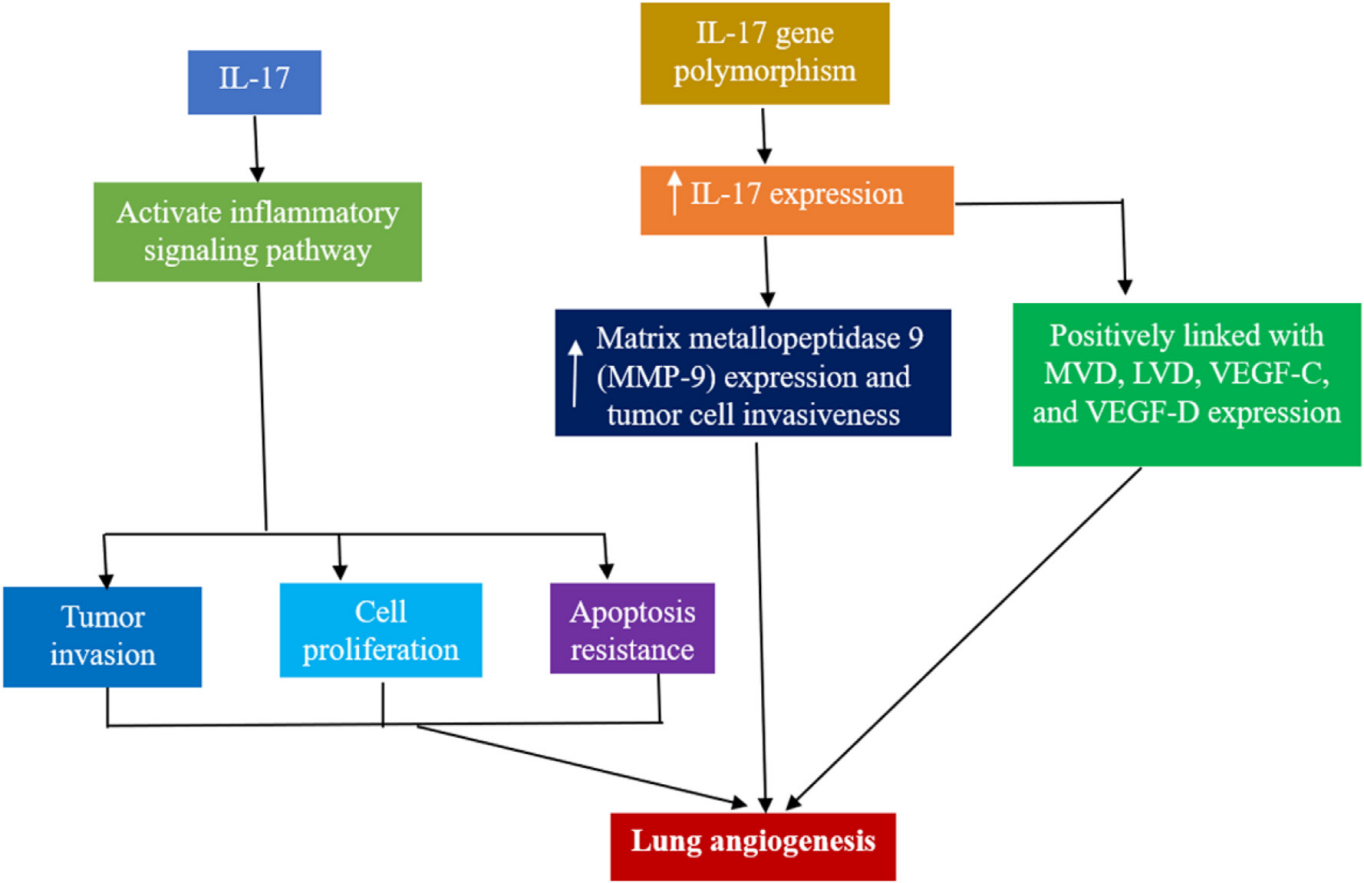
Mechanism of IL-17 in lung angiogenesis. IL-17 activates inflammatory signaling pathways and over-expresses MMP-9 to stimulate lung angiogenesis. IL: Interleukin; LVD: Lymphatic vessel density; MMP: Matrix metallopeptidase; MVD: Microvessel density; VEGF: Vascular endothelial growth factor.

IL-17, an inflammatory cytokine, is involved in chronic inflammatory and autoimmune diseases, as well as in inflammation-associated tumors. Research suggests that IL-17 helps LC spread by encouraging tumor angiogenesis, cell proliferation, and apoptosis inhibition, as well as by activating inflammatory signaling pathways. Additionally, through IL-17 overexpression, owing to MMP-9 expression and tumor cell invasiveness, IL-17 gene polymorphism exacerbates LC risk. These outcomes support IL-17 involvement in the growth of LC.^63^ When human LC tissues were immunohistochemically stained for CD31, CD34, and IL-17, a positive correlation was observed between tumor microvessel density (MVD) and increased IL-17 expression.^64^ Further investigations revealed that enhanced IL-17 production was favorably linked to high micro-lymphatic vessel density (LVD) and VEGF-C and VEGF-D expression.^65^ Numerous studies have shown that VEGF and its receptor family members are involved in the way that IL-17 stimulates angiogenesis or lymphangiogenesis. Brussino et al.*^66^* revealed a correlation between VEGF-A expression and IL-17 levels in patients with NSCLC.^67^ Serum IL-17 levels in patients with LC positively correlated with VEGF-A expression.^68^ Based on studies conducted on human NSCLC specimens, IL-17 is linked to the metastatic potential of lymph nodes and IL-17RA.^69^ IL-17 was found at a much higher level in the cerebrospinal fluid of patients with LC who had brain metastases than in those who did not, indicating a potential major function of IL-17 in brain metastasis of LC. The likelihood of brain metastasis is markedly increased in patients with LC with high serum IL-17 levels. Multiple studies have demonstrated that IL-17 is an essential prognostic and diagnostic indicator of LC.^63^ Meta-analyses revealed a high correlation between IL-17 overexpression and advanced-stage NSCLC (III/IV), overall survival (OS), as well as disease-free survival (DFS), suggesting that IL-17 promotes the growth and progression of NSCLC.^70^^,^^71^ Chen et al. identified IL-17 in human NSCLC tissues via an immunohistochemical technique.^72^ They noticed a strong correlation between patients’ clinical and pathological characteristics (including smoking status and TNM staging), as well as LVD, OS, DFS, and high IL-17 expression.^65^ Moreover, single- and multivariate analyses revealed that IL-17 was the only determinant of OS and DFS in patients with NSCLC. Wang et al. found a significant relationship between the average survival of patients with NSCLC and elevated IL-17 expression.^73^ Figure 7 illustrates the mechanisms by which IL-17 contributes to lung angiogenesis.

### Interleukin-18

Macrophages and a variety of innate immunological or non-lymphoid cells produce IL-18, an important pro-inflammatory cytokine in the inflammasome platform.^73^ Although it can be highly prevalent in patients with tumors, its association with the emergence of human cancers is unclear. Antibody-drug conjugates (ADCs) NSCLC-derived tumor cells produce IL-18, which is a key factor in driving type 1 (IFNγ-producing) responses of CD8C T-cells with high levels of IL-18 receptor (IL-18R), which are balanced by dysfunctional T-cells with low levels of IL-18R in ADC NSCLC. IL-18 may accelerate tumor growth, especially in the absence of Th1-like cytokines.^74^ There had been a noteworthy positive correlation seen in the TUM district (tumor tissue) between the concentrations of IL-18 and traditional Th1-associated cytokines, such as IFNγ, TNF-α, and IL-12. In a cohort of Iranian patients with LC and healthy controls, Farjadfar et al. examined three single nucleotide polymorphisms (SNPs) at locations – 656 (G/T), – 607 (C/A), and – 137 (G/C) within the IL-18 promoter. They found that in individuals with LC, the CA and AA genotypes, as well as the A allele at IL-18 gene position −607, were much more prevalent.^75^ They discovered a link between LC and the −607 polymorphisms, which could be due to the destruction of the potential cyclic adenosine monophosphate (cAMP)-sensitive element-binding protein location by the A allele, as well as a consequent decrease in IL-18 expression.^76^ IL18 levels in patients with NSCLC and SCLC were considerably higher than those in healthy smokers and non-smokers (*P* = 0.0001 and *P* = 0.001, respectively). Although patients with SCLC had higher sputum IL-18 levels than patients with NSCLC, this difference was not statistically significant.^10^ Previous studies have shown that patients with certain malignancies, particularly LC, have higher serum IL-18 levels. The IL-18 levels did not differ significantly between patients with NSCLC and those with SCLC. Additionally, the sputum supernatants of patients with NSCLC demonstrated a substantially greater proportion of VEGF/IL-18 than those of patients with SCLC, which is likely indicative of a more robust angiogenic effect in NSCLC.^76^

### Tumor necrosis factor-alpha

TNF*-*α is an important angiogenic cytokine that promotes angiogenesis by influencing the expression of other proangiogenic factors and increasing endothelial cell proliferation.^77^ TNF-308 and -238 polymorphisms significantly influence the prevalence of NSCLC, as demonstrated by the findings of a prior investigation that addressed their connection to LC susceptibility. The incidence of the infrequent TNF-α-308 A allele was considerably higher in patients with LC than in controls, while the frequency of the −238 A allele was considerably lower.^78^ In malignant tumors, such as LC, TNF-α is a major mediator that exhibits both antiangiogenic and tumorigenic actions.^79^ EBC testing is a completely painless technique for determining the pathophysiology of the airways in lung illnesses.^78^ The levels of 8-isoprostane, VEGF, and TNF-α in EBC and serum of patients having primary lung carcinoma were appraised in a prior study. They discovered that only TNF-α was higher in the EBC of patients with lung carcinoma when compared with controls; however, all three biomarkers are increased in the serum of individuals with LC.^93^ In accordance with previously noted greater concentrations of this biomarker both *in vitro* and *in vivo*, patients with LC had raised serum TNF-α levels.^79^ Alveolar macrophages, reactive alveolar pneumocytes, along luminal bronchial epithelial cells all showed immunopositivity for TNF-α, TNF-β, TNF-R1, as well as TNF-R2 in the nonneoplastic lung tissue. They discovered considerable co-expression of TNF-α, TNF-β, TNF-R1, and TNF-R2 in NSCLC.^80^

### Interferon-gamma

IFN-γ plays a vital role in adaptive as well as innate immune responses to fight off infections caused by viruses, bacteria, and protozoa. Although IFN-γ can promote tumor growth, several cancers have been treated clinically with it, indicating the contradictory role of IFN-γ in controlling antitumor immunity.^81^ IL-6 appears to be associated with well-known prognostic variables such as malnutrition, performance status, and severe illness. Consequently, multivariate analysis was conducted in a previous study to determine which variables had distinct prognostic values. In contrast to the Karnofsky index and malnutrition—which were likely excluded due to their strong association with elevated IL-6 and reduced IFN-γ—age and severe diseases such as lung cancer (LC) retained their prognostic significance.^49^ In patients with LC, there are no associations between ζ chain expression and IFN-γ production. The survival of patients with SCLC or NSCLC was not significantly impacted by ζ chain expression. There is no correlation between the possibility of survival and the median percentage of T or NK cells co-expressing IFN-γ in the two groups of patients with LC.^82^ Compared with healthy people, recent investigations showed considerably decreased levels of IFN-γ in whole blood cell cultures from patients with LC. The substantial variability, even in healthy volunteers, may have contributed to the reduced proportion of CD3+/IFN-γ+ cells in patients with LC relative to controls, though the variations were not statistically significant.^83^ Furthermore, no noticeable changes in IFN-γ secretion levels were identified between patients with SCLC and NSCLC.^84^ STAT1C had no appreciable impact on LC cell survival or proliferation when used alone or in combination with IFN-γ. A previous study showed that STAT1C expression alone causes only minor changes in IFN-γ-induced gene expression. Several pro-inflammatory gene products and chemokines controlled by IFN-γ/STAT1C signaling were found using oligonucleotide gene arrays and quantitative polymerase chain reaction (qPCR). These findings imply that IFN-γ and STAT1 have a large influence on pro-inflammatory gene expression rather than cell proliferation, survival, or both in non-small lung cells.^85^

### Interleukin-4

IL-4, IL-13, and STAT-6 are recognized for their roles in modulating immune functions. IL-4 and IL-13 are pivotal in controlling immune activity in the pulmonary context. These cytokines may cause structural changes in lung tissues by activating a variety of immune cells.^86^ Moreover, IL-4 and IL-13 are known to initiate the development of immunoglobulin E (IgE) in B cells and drive the differentiation of Th2 cells, playing key roles in allergic reactions within respiratory pathways.^87^

**Figure 8:**
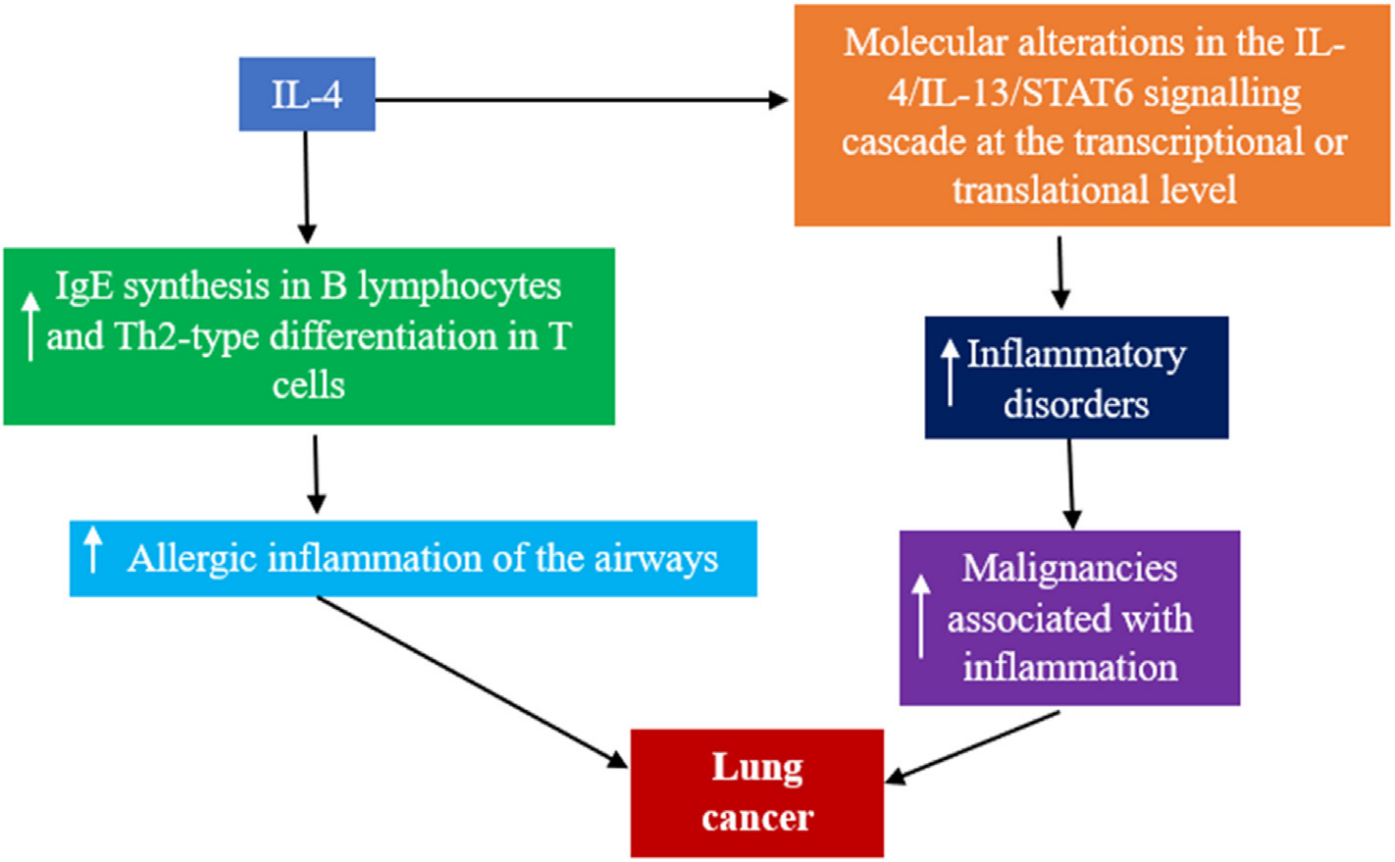
Correlation of IL-4 with LC. IL-4 initiates malignancies associated with inflammation by modulating immune cell action and lung cancer development. IgE: Immunoglobulin E; IL: Interleukin; LC: Lung cancer; STAT: Signal transducer and activator of transcription; Th: T-helper.

Previous studies have indicated that IL-4 inhibits the progression of abnormal cell growth in LC.^88^ Alterations in the genetic and protein expression levels of the IL-4/IL-13/STAT-6 pathway can significantly affect the development of inflammatory diseases and inflammation-related cancers, including LC.^86^ Figure 8 illustrates the potential link between IL-4 and LC progression. A notable percentage of NSCLC samples exhibited high levels of IL-4, IL-13, and STAT-6, suggesting a correlation with the disease.^88^

Research has also highlighted the influence of NSCLC subtypes on IL-4 mRNA levels, emphasizing the diverse effects of cytokines across the tumor microenvironment, both promoting and inhibiting tumor immunity and growth.^86^ Interestingly, IL-4 has been suggested to slow the proliferation of solid tumor cells, especially in LC, and may hinder tumor angiogenesis. Additionally, IL-4's interaction with CAFs, which is crucial for cancer progression and metastasis, has been documented.^89^

Overexpression of the IL-4 receptor (IL-4R) in LC cells has been investigated as a potential tissue-specific molecular marker.^90^ Both IL-4R and IL-4 are predictive markers at the protein level in various epithelial cancers, particularly LC.^91^ A previous study observed a significant difference in the prevalence of C/C + C/T genotypes between female patients with SCC and controls, suggesting that the C allele of the IL-4 gene's rs2243250 polymorphism might be linked to a greater risk of SCC among Chinese women.^92^

### Interleukin-5

The cytokine IL-5 generated by T-cells is crucial for the emergence of eosinophilia and is particularly effective in stimulating this cell type. A patient with NSCLC, leukocytosis, eosinophilia, high blood IL-5 levels, and a lymph node was identified in a previous study.^93^ Intracellular IL-5 was overexpressed in the excised original tumor, as determined by immunohistochemistry. Normal lung tissue stained with no antibody or the control technique revealed no intercellular IL-5 expression. IL-5 production is closely correlated with the tumor, and its removal causes the serum IL-5 levels to return to normal.^94^

### Interleukin-10

Various cells, including those in healthy and malignant tissues, generate IL-10, which has important anti-inflammatory properties. This cytokine is associated with several pathological and physiological events, including cancer progression, graft tolerance, and autoimmune conditions. Elevated levels of IL-10 in the serum and surrounding tumors have been detected in multiple types of cancers, including LC, suggesting the role of IL-10 in modulating tumor-related immune responses.^78^

Studies have indicated that individuals with NSCLC exhibit higher serum IL-10 levels than healthy individuals, with even higher levels detected in those with metastatic NSCLC.^95^ In 2005, Shih et al.*^78^* reported a significant association between NSCLC occurrence and genetic variation in the IL-10 genes. Tumor-associated macrophages (TAMs), which constitute a substantial part of the tumor mass, are believed to be instrumental in the co-evolution of tumors and their surrounding environment, as well as carcinogenesis.^96^

IL-10's role as a key immunosuppressive cytokine in cancer is well established. In NSCLC, increased IL-10 expression by TAMs correlates with more advanced stages of the disease, the presence of lymph node metastases, pleural invasion, lymphovascular spread, and lower differentiation of tumor cells, underscoring the significance of TAM-derived IL-10 in disease progression.^97^ IL-10's ability to suppress macrophage function may facilitate tumor evasion during immune detection, contributing to the advancement of LC.

Furthermore, greater amounts of IL-10 in the serum or tumor tissues have been linked to a reduced survival rate in patients with LC, highlighting the potential impact of IL-10 on cancer development. Cytokines are linked to angiogenic factors that are known to promote tumor growth in NSCLC, including VEGF and angiopoietin.^98^ IL-10's impact on the prognosis of LC has been documented, with studies showing that lung tumor nodules secrete significantly more IL-10 than typical lung tissue.^99^

While tumor cells release IL-10, they stimulate IL-10 production in lymphocytes via a PGE2-mediated mechanism.^100^ Consequently, T cell-derived IL-10 is considered a predominant contributor to the cytokine milieu surrounding lung tumors. Research on IL-10 mutant mice by Sharma et al.*^101^* suggested that these mice could produce increased levels of IL-10 both locally at the tumor site and systemically in the spleen.

### Transforming growth factor-beta

TGF-β is a cytokine with multiple roles, including the facilitation of tumor progression. Studies suggest that upregulation of TGF-β by tumors can help them evade immune detection, potentially leading to increased metastasis and recurrence.^102^ TGF-β protein expression has been linked to patient survival rates in NSCLC, suggesting that the protein plays a role in disease onset.^103^ High levels of TGF-β protein have been noted in patients with NSCLC at later stages.

Research indicates that TGF-β may contribute to creating an environment conducive to tumor growth, significantly influencing the onset and advancement of cancer.^104^ While normal epithelial cells may experience reduced growth in the presence of TGF-β, cancer cells often exhibit resistance, continuing to proliferate. This resistance is evident in LC cell lines, which show expression of TGF-β genes, mRNA, and proteins, suggesting a robust response that promotes cellular proliferation. Li et al. in 2019 reported a notable link between increased TGF-β levels and disease progression in patients with LC, with higher TGF-β levels associated with poorer survival outcomes in NSCLC.^104^ Additionally, prognostic indicators for LC include biomarkers such as miRNAs and long non-coding RNAs (lncRNAs). Emerging research points to the regulation of TGF-β signaling pathways by these RNA molecules, which can influence the migration, invasion, and metastasis of lung tumors.^102^

TGF-β is comprised of three isoforms – TGF-β1, TGF-β2, and TGF-β3 – and is recognized for its complexity and versatility as a cytokine. TGF-β facilitates the invasion, migration, and metastasis of LC.

### C-reactive protein

CRP is an acute-phase reactant and systemic indicator of chronic inflammation produced in response to various forms of tissue damage, including infections, physical trauma, heart attacks, surgical procedures, and cancerous growth.^105^ In 2012, Zhou et al. analyzed the link between CRP, IL-6, and LC risk and found a modest positive link between CRP levels before diagnosis and the likelihood of developing LC.^106^ Individuals who had quit smoking for as long as 15 years still had a heightened risk of LC if their CRP levels were elevated, although this was not the case with AC.^107^ However, high CRP levels were significantly associated with the risk of developing lung SCC and SCLC.^108^

In the context of LC, CRP levels can be erroneously high owing to infections that may occur during disease progression, often leading to increased morbidity and mortality.^106^ Elevated CRP levels before treatment have been shown to adversely affect the prognosis of patients with NSCLC.^109^ In 2013, Guo et al. conducted a meta-analysis that linked high blood CRP levels with a greater risk of cancer, particularly LC.^110^ However, it remains unclear whether elevated CRP is a symptom of the tumor or a causative factor.^111^ CRP has been identified as an inflammatory marker that is elevated in NSCLC, and its levels correlate with tumor size and grade but not with NSCLC subtypes, indicating a potential indicator of poorer prognosis and survival outcomes.^112^

### Insulin-like growth factor-1

**Figure 9:**
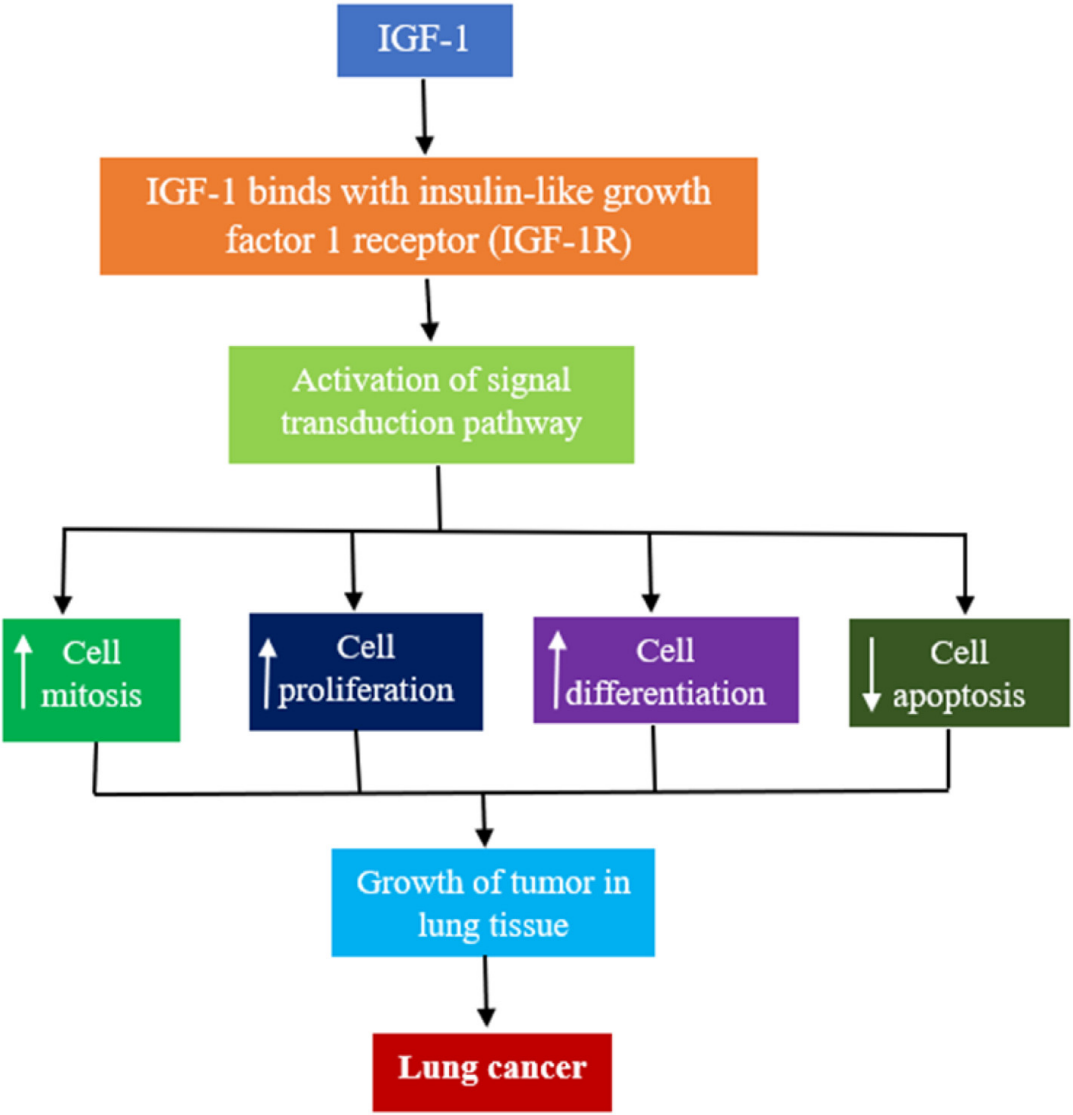
Mechanistic view of IGF-1 in LC: IGF-1 stimulates uncontrolled cell division and differentiation and downregulates cell apoptosis to promote tumor in the lung tissue and LC development. IGF: Insulin-like growth factor; IGF-1R: Insulin-like growth factor-1 receptor; LC: Lung cancer.

Cancer can occur because of disruptions in the cell cycle, unregulated cellular growth, and evasion of programmed cell death. The IGF family has been implicated in these processes as it can trigger cellular signaling pathways that promote cell division, growth, and differentiation while also inhibiting apoptosis through receptor interactions.^16^ This action is believed to play a significant role in the onset and progression of tumors. Specifically, IGF-1 has been spotlighted for its role in LC pathogenesis [Figure 9].^113^

A strong correlation between serum levels of IGF-1 and its binding protein, IGFBP-3, has been observed in individuals with LC, suggesting a link to disease severity. In advanced stages of NSCLC, higher plasma concentrations of these proteins are associated with more favorable outcomes.^114^ Laboratory studies have further suggested that IGF-1 may encourage the proliferation and invasion of lung cells, suggesting its contribution to the malignant growth of NSCLC.

The effects of growth hormones on cell proliferation are largely mediated by IGF-1. A 1999 study by Yu et al.^115^ found a connection between increased plasma IGF-1 levels and a heightened risk of developing LC. The ratio of IGF-1 to IGFBP3, the latter being the predominant carrier of IGF-1 in the bloodstream, has been proposed as an indicator of IGF-1's biological availability and may serve as a prognostic marker for the effectiveness of chemotherapy in NSCLC.^116^ In 2013, Wang et al. revealed that IGF-1 expression was significantly elevated in NSCLC compared with benign lung conditions, with its upregulation potentially accelerating the malignant transformation of LC cells, thereby enhancing their proliferative capacity.^117^

### Soluble tumor necrosis factor receptors

CgA is a specialized acidic glycoprotein that is prevalent within the secretory granules of neuroendocrine cells, including those in the adrenal medulla and various neurons. It belongs to a group of proteins that are regulated by secretion. In patients experiencing heart failure, CgA levels in the bloodstream can significantly increase, a change that is associated with TNF-α as well as its soluble receptors (sTNF-Rs), offering important prognostic information. In the case of NSCLC, research has shown that the serum concentrations of TNF-R1 and TNF-R2 are substantially higher than those in healthy individuals, as evidenced by statistical analysis (Wilcoxon test results: *Z* = −9.12 [*P* < 0.0001] for TNF-R1 and *Z* = −4.11 [*P* < 0.0001] for TNF-R2).^118^

### Transient receptor potential cation channel, subfamily M, member 7

Ion channels have been recognized as contributors to the onset and advancement of cancer owing to their altered expression in cancerous tissues and cell lines, which differs from their expression in healthy counterparts. These channels are implicated in cellular processes, such as proliferation, evasion of apoptosis, metabolic shifts, new blood vessel formation, and increased mobility of tumor cells. The TRPM7 channel, a nonselective cation channel that allows the entry of both calcium (Ca2+) and magnesium (Mg2+), is overexpressed in various malignancies, including those of the breast, prostate, pancreas, and ovaries. However, its specific expression pattern and influence on LC remain unclear.^119^^,^^120^

In 2018, Liu et al. highlighted the significant overexpression of TRPM7 in LC tissues and cell lines and noted its correlation with poor patient outcomes.^16^ Pathological variables, including tumor size, Ki67 proliferation index, and Scarff-Bloom-Richardson (SBR) grade, have all been positively associated with TRPM7 expression. Supporting this, Dhennin-Duthile et al. found that TRPM7, along with other TRP channels, such as TRPM8, is highly expressed in breast cancer cells and tissues.^120^

In LC, the TRPM7 expression is aberrant. It is found at high levels in lung AC and SCC, in contrast to its minimal expression in healthy lung alveoli. The mRNA levels of TRPM7 are greater in lung carcinoma tissues than in noncancerous tissues. Additionally, LC cell lines, such as SPCA-1, NCI–H520, SK-MES-1, A549, and 95D, exhibited higher levels of TRPM7 protein than the normal lung epithelial cell line, 16-HBE.^16^

### Urokinase plasminogen activator/plasminogen activator inhibitor type 1

Cancer metastasis is significantly affected by uPA. The PA inhibitors PAI-1 and PAI-2 are key regulators of uPA activity and are capable of binding rapidly to free-floating or receptor-bound uPA. PAI-1 is the primary physiological suppressor of uPA and is secreted as an active protein that stabilizes upon binding to vitronectin in the extracellular matrix (ECM). The formation of an inactive complex between active PAI-1 and uPA is essential for regulating enzymatic activities.^121^

Analysis of tissue specimens from 99 individuals with lung AC with an average survival period of 25 months demonstrated the predictive value of PAI-1. The study is the first to measure the uPA-PAI-1 complex in a cancer type other than breast cancer, allowing for the analysis of its correlation with clinical outcomes in LC.^121^

Further investigation confirmed the presence of uPA and its receptor, uPAR, in LC tissues. High uPAR levels in squamous cell LC extracts have been linked to poor prognosis. In addition, elevated plasma uPAR concentrations have been observed in patients with NSCLC.^122^ A pioneering study by Yang et al. established a correlation between uPA levels and medical features in patients with NSCLC and found that patients with stage IV tumors exhibited higher uPA levels, particularly in cases of SCC.^123^

### Matrix metalloproteinase-2

MMPs, specifically MMP-2 and MMP-9, facilitate angiogenesis, tumor growth, and cancer spread. Studies have shown that these enzymes are present in higher amounts in malignant tumors than in benign tumors. Ongoing research on MMP levels in LC has led to the advancement of new methods aimed at inhibiting these enzymes. Notably, increased net activity of MMP-2 has been observed in patients with NSCLC, and this activity appears to be closely linked to the TNM classification of tumors. Furthermore, the extent of lymph node metastasis was directly associated with the net activity of MMP-2.^14^ The presence of MMP-2 has also been implicated in the initiation of new blood vessel formation, as tumors expressing MMP-2 tend to have a higher number of capillaries at the interface with tumor cells.^124^ In a study by Wadowska et al. in 2021, the expression of TIMP-1 was notably higher in SCLC, particularly in the common illness subtype (*P* < 0.05).^125^ Similarly, MMP-2 levels were notably higher in stage 2 NSCLC, particularly in the AC subtype, also with a *P* value < 0.05. These outcomes are supported by multiple studies, suggesting that an increase in MMP-2 expression could be an important indicator of NSCLC progression.

### Matrix metalloproteinase-9

MMPs play a pivotal role in the degradation and remodeling of the ECM and basal membrane (BM), which are critical steps in cancer progression and metastasis. MMP-9 is an enzyme crucial for ECM breakdown. Located in the 20q12-13 region of the human chromosome, MMP-9 has been implicated in various cancer types.^126^ MMP-9 expression has been identified in the urine, serum, plasma, and malignant tissue of patients.^123^ Research on the genetic variations of MMP-9-1562C/T has indicated that those with the T allele may have a decreased likelihood of developing LC. In contrast, those with the C allele may be at a higher risk. Specifically, the CC genotype of MMP-9-1562C/T has been linked to a higher risk of NSCLC in patients in Southern China, with serum concentrations of MMP-9 measured in the control groups.^126^ Additional evidence for MMP-9's role in NSCLC includes higher collagenase activity in macrophages from the bronchial lavage fluid of patients with NSCLC, higher mRNA expression, and higher protein levels, as determined by immunohistochemistry. Moreover, the presence of mesenchymal stem cells (MSCs) has been observed to increase the secretion of MMP-9 by lung AC cells. High MMP-9 expression in patients with lung AC correlates with poor survival outcomes.^123^

### Matrix metalloproteinase-13

Collagenase, or MMP-13, is an important enzyme involved in the rapid restructuring of the ECM and plays an essential role in the degradation of various collagen fibrillar structures, including collagen I, II, III, and VII. MMP-13, comprising 10 exons and nine introns, is located on chromosome 11q22 and covers approximately 12.5 kB. The heightened expression of MMP-13 observed in diverse tumor types indicates its potentially strong association with cancer progression, metastasis, and unfavorable prognosis across several cancers. MMP13 levels have been detected in the serum of patients with NSCLC as well as in healthy controls.^126^ Li et al. revealed a link between MMP13 genetic variations and the risk of LC development using both non-model-based approaches and recessive genetic models.^127^ Further research suggests that increased MMP13 expression is associated with the invasion, spread, and recurrence of NSCLC. Individuals carrying the MMP-9 CC as well as MMP13 GG genotypes have a substantially higher risk of developing NSCLC, particularly in the Southern Chinese population. This finding is supported by the measurement of MMP-9 and MMP13 levels in the blood, which underscores the potential role of these genes as oncogenic factors in NSCLC.^126^ Additionally, the cytokine TNF-α has been implicated in the advancement of LC by activating the ATM pathway as well as upregulating MMP-13 expression.^128^

### *Tissue inhibitors of metalloproteinase-1* and *tissue inhibitors of metalloproteinase-2*

TIMP1 is an element of the protein series that inhibits the action of MMPs, which are crucial for the maintenance and alteration of the ECM. There are four distinct members of the TIMP family: TIMP1, TIMP2, TIMP3, and TIMP4. TIMP1 and TIMP2 promote cell proliferation, and TIMPs can stimulate growth independent of their MMP-inhibiting functions. Research has shown that TIMP1 levels are elevated in various tumor types, especially NSCLC, compared with those in healthy tissues.^129^

TIMP-1 is expressed more abundantly in LCs than in other types of tumors, including colorectal cancer and related liver metastases. In contrast, neither benign nor healthy lung tissue exhibited increased TIMP-2 mRNA levels.^126^ Studies on LC cell lines from different histological backgrounds have examined TIMP-1 and TIMP-2, natural inhibitors of gelatinases, and found that their gelatinolytic activity aligns with the clinical patterns of spread observed in SCLC.^129^

### Monocyte chemoattractant protein-1

Human bronchial epithelial cells are known to generate a range of pro-inflammatory mediators, including MCP-1, IL-8, and growth-related oncogene α (GROα). These cytokines play crucial roles in eliminating pathogens, drawing inflammatory cells to sites of infection, promoting mucin production, and aiding tissue remodeling.^130^ MCP-1, a member of the CC chemokine superfamily, is particularly important for monocyte attraction and activation during angiogenesis and acute inflammatory response. In the human lung, MCP-1 is found in various cell types, including macrophages and endothelial, bronchial epithelial, and smooth muscle. Studies have shown that LC cell lines, such as H1299 and A549, produce higher levels of MCP-1 and other inflammatory mediators like RANTES, ENA-78, GRO, GROα, IL-8, VEGF, CXCL16, and MMP-9, compared with non-cancerous BEAS2B cells. Although IL-8 has been suggested to be a significant angiogenic factor in NSCLC, research on the clinical implications of MCP-1 and IL-8 in LC progression is limited. However, A549 LC cells expressed both MCP-1 and IL-8. Patients with localized LC exhibit elevated levels of these cytokines compared with healthy individuals, and those with bone metastases show even higher levels. Consequently, MCP-1 and IL-8 can potentially serve as biomarkers of tumor progression, particularly in the context of bone metastasis.^131^

### Vascular endothelial growth factor

VEGF and its corresponding receptors (vascular endothelial growth factor receptors [VEGFRs]) are vital for the growth, movement, and invasive capabilities of endothelial cells. Human VEGF, which spans eight exons and seven introns, is located on chromosome 6p21.1.^132^ VEGF is a key angiogenic molecule in NSCLC, with high expression or significant angiogenic activity observed in approximately 30–40% of NSCLC cases.^133^ Elevated levels or overexpression of VEGF in the serum have been noted in both NSCLC and SCLC. VEGF functions through its interaction with the tyrosine kinase receptors VEGFR1 and VEGFR2, which are predominantly found in endothelial cells, many non-endothelial cells, and cells from several types of human cancers, particularly LC.^132^ Research indicates that NSCLC cell lines express and activate VEGFRs, suggesting the presence of autocrine signaling mechanisms. Similarly, human SCLC cell lines express VEGF and phosphorylate VEGFR2, suggesting autocrine support of cellular functions in SCLC.^133^, ^134^, ^135^ The expression of hypoxia-inducible factor (HIF) is a key regulator of VEGF function in many cancers, including LC. Hypoxic conditions markedly increase VEGF mRNA levels due to sustained activation of HIF-1 in LC cell lines with metastatic potential.^136^ Furthermore, the transcription factor SP-1 is essential for boosting VEGF expression in NSCLC cells.^130^ The transcription factor EGR-1 also contributes to VEGF production in NSCLC cells via both HIF-1α-dependent and independent pathways. Experimental mouse models have shown that the continuous expression of VEGF in a specific LC cell line promotes tumor growth.^137^

### Soluble intercellular adhesion molecule-1

Angiogenesis, the creation of new blood vessels, and the growth of tumor cells are all linked to sICAM-1.^138^ Enhanced sICAM-1 levels have been linked to the progression and dissemination of various cancers. Studies have revealed that patients with LC exhibit significantly higher sICAM-1 levels in their bloodstream than individuals without LC.^139^ Research conducted by Benedicto et al. implied that surrounding tissues or metastasizing organs may also emit sICAM-1.^140^ The production of sICAM-1 can be stimulated by a range of cytokines and growth factors secreted by LC cells, potentially explaining the increased sICAM-1 levels observed in patients with LC.^141^

The role of sICAM-1 in angiogenesis in NSCLC was indirectly supported by Qian et al., who found a strong correlation between baseline serum sICAM-1 and VEGF levels.^20^ This indicates that sICAM-1 significantly affects tumor growth and metastasis. ICAM-1 expression is predominantly found in the bronchial and alveolar epithelial cells of LC tissues and is consistent across different histological types. Because ICAM-1 is produced by tumor cells, there is a notable association between serum ICAM-1 levels and its expression in tumor tissues.

Clinically, the initial levels of sICAM-1 in the blood may act as a predictive marker for both NSCLC and SCLC.^139^ Grothey et al. studied ICAM-1 expression in tumor cells via immunohistochemical examination in 20 patients with NSCLC and examined sICAM-1 blood levels in 51 patients with NSCLC and 40 normal controls (both smokers and non-smokers). Their findings revealed that high ICAM-1 expression, which is common in SCLC and all histological subtypes of NSCLC, enhances the metastatic potential of malignant tumors. Moreover, individuals with NSCLC had higher blood sICAM-1 levels than controls.^22^^,^^142^

### Myeloperoxidase

MPO, an enzyme found in neutrophils and monocytes, promotes LC development by stimulating various procarcinogens, including benzo[a]pyrene derivatives, 4-aminobiphenyl, and arylamines. Research by Schabath et al. indicated that individuals with no less than one.^23^ A allele of MPO experienced a lower risk of LC, with an OR of 0.52 and a 95% CI of 0.30–0.90. London et al. first reported a link between the MPO variant allele and LC risk, noting an OR of 0.30 and a 95% CI of 0.10–0.93. Further analysis showed that Caucasian men with G/A or A/A genotypes experienced a substantially lower LC risk (OR = 0.55; 95% CI = 0.36–0.84), a pattern not observed in women.^143^ Individuals with the A/A genotype are more prone to SCC and AC.^25^

The role of MPO in biotransformation involves the conversion of B[a]P-7,8-diol in tobacco smoke into carcinogenic benzo[a]pyrene diol epoxide. Despite the lower transcriptional function of the variant allele, B[a]P-7,8-diol was active at lower enzyme levels, which may reduce the risk of LC. This safeguarding effect was primarily observed in men, young individuals, and heavy smokers. These outcomes offer epidemiological evidence for the role of MPO in the metabolic release of carcinogens from tobacco smoke and their impact on LC risk.^144^

### Emerging treatments targeting inflammatory pathways in lung cancer

Inflammation plays a pivotal role in the progression of LC, the proliferation of lung tumors, angiogenesis, and immune invasion.^145^ To improve patient outcomes, emerging treatments have focused on modulating certain inflammatory mediators.^146^ Monoclonal antibodies against IL-6, including tocilizumab, and TNF-α inhibitors, such as infliximab, are examples of cytokine-targeting drugs that have shown promise in mitigating inflammation linked to tumors. Furthermore, small-molecule inhibitors targeting the Janus kinase (JAK)/STAT and NF-κB pathways are being investigated to suppress cytokine-driven tumor growth.^147^ Immunotherapy, specifically with immune checkpoint inhibitors, such as pembrolizumab and nivolumab, has revolutionized LC treatment by enhancing T cell-mediated responses and indirectly modifying inflammatory pathways.^148^ Additionally, treatments such as colony-stimulating factor-1 receptor (CSF-1R) inhibitors that target TAMs alter the tumor microenvironment and reduce immunological suppression.^149^

IL-6 and IL-10 are two important inflammatory mediators that have attracted considerable attention in drug development.^150^ Pro-inflammatory cytokine IL-6 is a potential target for inhibitors, such as tocilizumab, because it stimulates the growth of tumors via the JAK/STAT3 and NF-κB pathways.^151^ The inhibition of IL-6 signaling slows tumor growth and improves the effectiveness of immunotherapy.^152^ On the other hand, IL-10, known for its immunosuppressive qualities, plays a dual role in LC.^153^ Although its overexpression can facilitate immune escape, IL-10-based therapies, such as pegilodecakin, have been studied for their potential to activate cytotoxic T-cells when combined with immune checkpoint inhibitors.^154^

Future treatment efforts will likely focus on selectively altering these cytokines to balance pro- and anti-inflammatory responses, improve medication efficacy, and reduce adverse effects. This study provides a comprehensive analysis of inflammatory mediators and their therapeutic potential, identifies key molecular targets that could potentially enhance existing treatments, and guides future research on LC.^155^

Despite the valuable insights gained from studies on the inflammatory pathways in LC, certain limitations must be addressed. Variability in sample sizes across studies is a major concern, as it may compromise the statistical power and generalizability of the findings. Many studies have relied on small cohorts, which limit their relevance to larger patient populations. Furthermore, the regional focus introduces additional possible bias since research conducted in specific regions may fail to account for genetic and environmental variables that influence inflammatory responses in LC. Differences in study design, such as retrospective analysis and heterogeneity in patient selection criteria, may have resulted in additional contradictions in the reported results. Furthermore, comorbidities, lifestyle, and treatment history, which are not always controlled, can influence cytokine levels and their prognostic significance. Finally, although preclinical and *in vitro* studies have provided molecular insights, their translation to clinical efficacy remains difficult. Addressing these constraints through larger, well-controlled, multicenter studies is critical for improving the therapeutic methods targeting inflammation in LC.

## Conclusions

This review provides a comprehensive summary of various factors that potentially influence LC development and progression. The diverse functions of pro-inflammatory cytokines, ILs, and certain factors, such as IGFs, sTNF-Rs, TRPM7 ion channels, uPA, MMPs, MCP-1, VEGF, sICAM-1, and MPO, contribute to the complexity of this disease. The findings of this study contribute to the existing literature by elucidating and expanding on the roles of inflammatory cytokines and other factors in LC progression. Key cytokines, such as TNF-α, IFN-γ, TGF-β, and various ILs and their regulatory roles in the proliferation, metastasis, and apoptosis of LC cells were highlighted. Furthermore, it identified the involvement of macrophages in NSCLC and the prognostic significance of markers such as CRP, IGFs, and MMPs. Moreover, this review included a thorough analysis of genetic variants, especially in MPO, and the combination of cytokine-mediated pathways with tumor angiogenesis and immune evasion mechanisms. Overall, this review provides a comprehensive molecular and immunological viewpoint, supporting the potential of targeted prognostic assessments and future therapeutic strategies in LC.

## Authors contribution

Md. Shalahuddin Millat: writing – original draft, formal analysis; Md. Mahmudul Hasan: writing – original draft, formal analysis; Mohammad Sarowar Uddin: writing – original draft, software; Md. Abdus Salam: writing – original draft; Md. Abdul Aziz: Writing– original draft, software; Irin Akhter: – original draft, data curation; Md. Saddam Hussain: data curation, conceptualization; Nor Mohammad: data curation, conceptualization; Farjana Afrin Tanjum: data curation; Md. Saqline Mostaq: validation, resources; Md. Ashiq Mahmud: investigation, data curation; Mohammad Nurul Amin: validation, resources, investigation, data curation; Mohammad Safiqul Islam: validation, supervision, project administration, conceptualization. All the authors have read and approved the final paper.

## Ethics statement

None.

## Data availability statement

The datasets used in the current study are available from the corresponding author on reasonable request.

## Declaration of generative AI and AI-assisted technologies in the writing process

The authors declare that generative artificial intelligence (AI) and AI assisted technologies were not used in the writing process or any other process during the preparation of this manuscript.

## Funding

None.

## Conflict of interest

The authors declare that they have no known competing financial interests or personal relationships that could have appeared to influence the work reported in this paper.
